# Ultrasound-Based Method for the Identification of Novel MicroRNA Biomarkers in Prostate Cancer

**DOI:** 10.3390/genes12111726

**Published:** 2021-10-28

**Authors:** Jessica Cornice, Daria Capece, Mauro Di Vito Nolfi, Monica Di Padova, Chiara Compagnoni, Daniela Verzella, Barbara Di Francesco, Davide Vecchiotti, Irene Flati, Alessandra Tessitore, Edoardo Alesse, Gaetano Barbato, Francesca Zazzeroni

**Affiliations:** 1 Department of Biotechnological and Applied Clinical Sciences (DISCAB), University of L’Aquila, 67100 L’Aquila, Italy; jessica.cornice@graduate.univaq.it (J.C.); mauro.divitonolfi@univaq.it (M.D.V.N.); monica.dipadova@univaq.it (M.D.P.); chiara.compagnoni@univaq.it (C.C.); daniela.verzella@univaq.it (D.V.); barbara.difrancesco1@univaq.it (B.D.F.); davide.vecchiotti@univaq.it (D.V.); irene.flati@graduate.univaq.it (I.F.); alessandra.tessitore@univaq.it (A.T.); edoardo.alesse@univaq.it (E.A.); francesca.zazzeroni@univaq.it (F.Z.); 2 Inno-Sol srl, Via della Ricerca Scientifica snc, ed. PP1, 00133 Rome, Italy; gaetano.barbato@uniroma2.it; 3 Department of Biology, School of Pharmacy, University of Rome Tor Vergata, 00133 Rome, Italy

**Keywords:** miRNAs, biomarkers, prostate cancer, ultrasounds

## Abstract

The detection of circulating microRNA (miRNA)-based biomarkers represents an innovative, non-invasive method for the early detection of cancer. However, the low concentration of miRNAs released in body fluids and the difficult identification of the tumor site have limited their clinical use as effective cancer biomarkers. To evaluate if ultrasound treatment could amplify the release of extracellular cancer biomarkers, we treated a panel of prostate cancer (PCa) cell lines with an ultrasound-based prototype and profiled the release of miRNAs in the extracellular space, with the aim of identifying novel miRNA-based biomarkers that could be used for PCa diagnosis and the monitoring of tumor evolution. We provide evidence that US-mediated sonoporation amplifies the release of miRNAs from both androgen-dependent (AD) and -independent (AI) PCa cells. We identified four PCa-related miRNAs, whose levels in LNCaP and DU145 supernatants were significantly increased following ultrasound treatment: mir-629-5p, mir-374-5p, mir-194-5p, and let-7d-5p. We further analyzed a publicly available dataset of PCa, showing that the serum expression of these novel miRNAs was upregulated in PCa patients compared to controls, thus confirming their clinical relevance. Our findings highlight the potential of using ultrasound to identify novel cell-free miRNAs released from cancer cells, with the aim of developing new biomarkers with diagnostic and predictive value.

## 1. Introduction

MiRNAs are ~22-nucleotide long noncoding sequences of RNA that are located across the genome, within an intron or untranslated region (UTR) of a coding gene [1]. Pri-miRNAs are transcribed from their genes in longer primary transcripts which are processed by two RNase III proteins—Drosha and Dicer—to form a functional miRISC complex that binds to the 3′ UTR of target mRNAs and induces their degradation and translational repression [2].

miRNAs were found to be highly stable in blood and other body fluids, where they circulate in a cell-free form, bound to other proteins, lipids, or lipoprotein or encapsulated in exosomes [3,4,5,6]. The development of specific high-throughput detection methods allowing miRNA detection in extracellular fluids, besides the fact that profiles of miRNAs were shown to be either downregulated or overexpressed across several cancer types compared to normal counterparts [7,8,9,10], has paved the way for serum miRNAs to be developed as biomarkers for early detection and monitoring of tumor evolution [5,11,12]. However, significant challenges remain, such as the low concentration of miRNAs released in the blood, especially in early-stage disease, and the difficult identification of biomarker release sites [13,14].

In recent years, ultrasound (US)-based techniques were proven to be useful tools that could advance the clinical application of miRNA-based biomarkers [15]. US-dependent cavitation changes the physiologic state of the cell membrane, increasing membrane permeability and establishing an enhanced bidirectional efflux that facilitates both uptake and release of low-molecular-weight molecules, like drugs, peptides, proteins, and nucleic acids [16].The applicability of ultrasound-based method for the amplification of circulating biomarkers was demonstrated in several in vitro and in vivo models, including soft tissue and xenograft models and tumors from patients [17]. Interestingly, it has been shown that the biomarkers release triggered by US treatment of subcutaneous and orthotopic xenograft tumors was not negatively affected by the presence of the more complex vasculature and structural density normally surrounding tumors in vivo [17]. Moreover, pre- and post-treatment biomarkers quantification analysis in patients who underwent routine Magnetic Resonance-guided–Focused Ultrasound Surgery (MRg–FUS) treatment of uterine fibroids showed a partial increase in protein biomarkers like Endothelin-1 and CA125 and increased levels of the microRNAs miR-21, miR-363, and miR-490 in a subgroup of patients [17]. The US regimen required for tumor ablation induces mechanical tumor tissue disruption, whose effects were deeply analyzed by Chevillet et al. in 2017 [18]. The study pointed out that, differently from the tissue liquefaction treatment, in mild heating regimens tissue architecture remains intact, with only some damaged surrounding tumor cells and petechial hemorrhage after the permeabilization treatment. As expected, the liquefaction regimen induced a massive release of microRNAs due to the mechanical destruction of cells and tissue structure. However, a similar effect, even if smaller, was produced with the permeabilization treatment that also increased the abundance of circulating microRNAs [18]. Therefore, this recent preliminary evidence showed that the application of an appropriate amount of ultrasonic energy to the tumor site promoted the release of specific biomarkers in the blood stream, thus suggesting that this minimally invasive method could be clinically useful to stimulate nucleic acid biomarker release from a specific tumor site of interest into the circulation, although further investigation is needed [17,18].

In this study, we investigated the potential of an innovative ultrasound-based prototype to improve the release of miRNAs in extracellular fluids, with the aim of identifying novel miRNA-based biomarkers that could be potentially used for prostate cancer (PCa) diagnosis and prognosis and for the monitoring of treatment response. PCa is a very heterogeneous disease, entailing quite different clinical behaviors, from clinically indolent to lethally aggressive [19]. Currently, the two most used screening tools for PCa are serum prostate-specific antigen (PSA) levels and digital rectal examination (DRE). Nevertheless, even if serum PSA detection has facilitated PCa diagnosis at early disease stages, the lack of cancer-specificity of this biomarker has resulted in a high false-positive rate, overdiagnosis, and consequent overtreatment [20]. Thus, the development of novel effective screening and prognostic biomarkers for managing PCa patients remains an unmet need. We provide evidence that US-mediated sonoporation amplified the release of miRNAs from both AD and AI PCa cells, allowing the identification of novel miRNAs related to PCa: miR-629-5p, miR-374-5p, miR-194-5p, and let-7d-5p. Bioinformatic analysis of miRNA molecular targets highlighted their role in PCa development and progression, while the analysis of their expression in publicly available datasets of PCa patients confirmed their relevance in clinical settings.

## 2. Materials and Methods

### 2.1. Cell Culture

LNCaP, DU145, PC3, and 22Rv1 PCa cell lines were purchased from the American Type Culture Collection (ATCC, Manassas, VA, USA). Cells were cultured in a humidified incubator in 5% CO2 at 37 °C. DU145 and PC3 cells were cultured in RPMI-1640 medium (#30-2001, ATCC) supplemented with 10% Fetal Bovine Serum (FBS), (certified, One Shot™ format, Thermo Fisher Scientific, Waltham, MA, USA); 22Rv1 cells were cultured in Dulbecco’s modified Eagle’s Medium (DMEM) with 10% FBS; LNCaP cells were cultured in RPMI 1640 with 10% FBS supplemented with 10 mM HEPES (#ECM0180D, Euroclone, Mlan, Italy) and 1 mM Sodium Pyruvate (#ECM0542D, Euroclone, Mlan, Italy). All media were supplemented with antibiotics (150 U/mL penicillin, 200 U/mL streptomycin) (#ECB3001D, Euroclone, Mlan, Italy) and 2 mM Glutamine (#ECB3000D, Euroclone, Mlan, Italy). All cell lines were routinely tested using a PCR Mycoplasma Detection Set (#6601, Takara). Cell line authentication (STR) was carried out.

### 2.2. Ultrasound Instrument and Treatment

Sonication was performed with Sonowell^®^ (Rome, Italy) [21], designed to operate on standard well plates. Our version has 4 flat transducers of 12 mm diameter, frequencies of 0.65, 1.0, 2.4, and 4.5 MHz, whose emission is controlled with a 4-channel generator/amplifier operating in parallel. The instrument is equipped with a thermostatic tank controlling the sample temperature kept constantly at 37 °C. A robotic x,y,z system provides the controlled well plate displacement on the transducers. The set-up is of the type well-on-transducer, with the plate inner-side bottom end calibrated to be 1 mm below the Near/Far Field plane (N/F Field) [22]. The geometry of the transducers positions the N/F Field plane at the same Z quote for all the frequencies, ensuring that all the samples experience the sonication wavelets at the maximum of the excitation profile, so that the sample solution is homogeneously sonicated in the Far Field. Protocols of sonication were set with the instrument software SonoWell Soft© (Inno-Sol, Roma, Italy), which allows the simultaneous use of 4, 2, or 1 transducers on the same well plate. Our experiments were optimized on Falcon 24-well plates. Calibrations of the acoustic pressure used in the experiments were performed using a needle hydrophone (0.5 mm) preamplifier and a DC coupler (Precision Acoustics, Dorchester, UK), monitoring the amplitude of the negative-peak detected signal with a Fluke Scopemeter 125 (Fluke, WA, USA). Calibration curves of mW/cm^2^ vs. acoustic pressure measured in kPa were obtained for all the transducers over the range of 0.010–6.0 W/cm^2^. PCa cells were plated in 24-well plates (Falcon) at the concentration of 150.000 cells/well. Cell layers were rinsed with PBS after either 24 (22Rv1, DU145, PC3) or 48 (LNCaP) h of incubation in complete media; then, fresh medium was completed with exosomes-depleted FBS to eliminate interference of RNA-containing exosomes from FBS, and protease inhibitors were added to avoid PSA degradation by intracellular proteases released following US treatment (#04693159001, Roche, Basel, Switzerland). Supernatant samples were collected before and after sonication from the same well; the incubation time of the untreated control cells was equal to the sonication period [17]. Top sealing films were applied to the plates, covered with an ultrasound coupling gel and a layer of phono-absorbent material. The frequency of 1 MHz was selected for the treatment of all cell lines, as previously reported [17]. DU145, PC3, and 22Rv1 cells were treated at Ispta 410.5 mW/cm^2^, 10% DC for 15 min, 30 min, and 1 h; LNCaP cells were treated with the same parameters at lower intensities (Ispta 307.9 mW/cm^2^) to avoid the detachment of the cell monolayer. Higher percentages were assessed to cause membrane disruption and morphological alterations. Samples were centrifuged for 30 min at 1500× *g* to remove the detached cells and debris and processed for protein and miRNA quantification. Readouts were performed after 15 min of US treatment to allow the resealing of cell sonication pores.

### 2.3. Homogeneity of Well Surface Coverage

Evaluation of the homogeneity of transducer wavelet transmission over the adherent cellular layer was carried out using DU145 cells plated at confluency in a 24-well plate and treated with US at 500 kPa acoustic pressure, 650 KHz, 1 MHz, 2,4 MHz and 4,2 MHz. Cells were stained with Crystal Violet (0.2%, #C0775, Sigma-Aldrich, Burlington, MA, USA), and ImageJ software (1.47v) [23] was used to evaluate the percentage of well surface coverage. The homogeneity of the detachment profiles was evaluated comparing images before and after sonication.

### 2.4. Protein Quantification, Total RNA Extraction, and Analysis

The culture media of PCa cells were analyzed for PSA concentration using a Human Prostate-Specific Antigen (PSA) ELISA kit (#EA100981, Origene, Rockville, MD, USA). PSA standards (1.56 ng/mL, 3.12 ng/mL, 6.25 ng/mL, 12.5 ng/mL, 25 ng/mL) were provided in the kit. Total RNA was isolated using the TRIzol Reagent (RiboEX™, Cat. No. 301-001, GeneALL^®^, Seoul, Korea). The isolated RNA was spectrophotometrically quantified, and equal amounts were used for cDNA synthesis using a High-Capacity cDNA Reverse Transcription Kit (#4368814, Applied Biosystems™, Thermo Fisher Scientific, Waltham, MA, USA). Real-time polymerase chain reaction (RT-qPCR) was performed by using the Luna^®^ Universal qPCR Mastermix (#M3003X, New England Biolabs, Ipswich, Massachusetts USA). Primers for prostate-specific antigen (PSA) spanning exon 1–2 were purchase from IDT (S: 5′-AACCAGAGGAGTTCTTGACCCC-3′, AS: 5′-GAACTTGCGCACACACGTC-3′).

### 2.5. MiRNAs Extraction 

miRNAs extraction from supernatants, collected before and after sonication, as carried out with the Plasma/Serum RNA Purification Mini Kit (#55000, NORGEN BIOTEK. CORP, ON, Canada) according to the manufacturer’s instructions. Briefly, supernatant samples were collected and centrifuged for 10 min at 1500× *g*, 4 °C, to remove detached cells and debris. Then, 5 μL of *C. Elegans* spike-in control miRNAs at a concentration of 5 fmol/μL, was added before the extraction of total miRNA. miRNA concentrations and quality were evaluated by using NanoDrop 2000 (Thermo scientific, Waltham, MA, USA). miRNA expression profiling was performed using TaqMan™ Advanced miRNA Human Serum/Plasma RT-qPCR array cards (#A34717, Life Technologies, Thermo Fisher Scientific, Waltham, MA, USA) for detection of up to 188 unique miRNAs in one serum/plasma sample. Endogenous and exogenous miRNA controls for the normalization of the data results are included in the array card. Data were collected at a 0.1 threshold value on an Applied Biosystems ViiA7 Real-Time PCR System. The Ct values were normalized to the mean of Ct values of cel-mir-39 and analyzed with software Expression Suite (Life Technologies, Thermo Fisher Scientific, Waltham, MA, 02451, USA). miRNA quantification in cell supernatants was measured by using TaqMan RT-qPCR. The results were standardized to the spike-in control *C. elegans* miRNAs (#000200). Taqman single-tube assays for miR-16-5p (#000391), miR-200c-3p, (#002300), miR-141 (#000463), miR-629-5p (#002436), miR-374a-5p (#000563), miR-194-5p (#000493), and let-7d-5p (#002283) were purchased from Thermo-Fisher (Waltham, MA, USA). 

### 2.6. Patient Dataset and Bioinformatic Analysis

Gene Expression Omnibus (GEO) GSE112264 dataset reporting serum miRNA profiles data in samples from 809 PCa patients and 41 healthy control volunteers was analyzed using GEO2R software (Version info: R 3.2.3, Biobase 2.30.0, GEOquery 2.40.0, limma 3.26.8). Gene Expression Profiling Interactive Analysis (GEPIA) web server was used for expression analysis of the *COL271A* gene in PCa (*n* = 492) and normal tissue (*n* = 152) from PCa patients and healthy control volunteers from the Cancer Genome Atlas Prostate Adenocarcinoma (TCGA-PRAD) and Genotype-Tissue Expression (GTEx) datasets. DIANA-miRPath 3.0 web server tool (http://diana.cslab.ece.ntua.gr/pathways/, accessed on 28 February 2021) was used to perform an enrichment analysis of the validated miRNA–gene interactions for each miRNA, followed by targeted-pathway analysis. The miRNET free software was used to generate miRNA–gene interactions network (https://www.mirnet.ca/, accessed on 1 March 2021). Please, refer to the data availability statement section for additional information.

### 2.7. Statistical Analysis

All data are presented as mean ± standard deviation (SD) unless stated otherwise. 

The statistical significance for two-sample comparisons was calculated using two-tailed Student’s *t*-test or two-tailed Mann–Whitney U-test, as appropriate. The statistical significance for multiple-sample comparisons was calculated using the Kruskal–Wallis test, as indicated. *p*-values < 0.05 were considered statistically significant. Analyses used GraphPad Prism version 6.0 for Windows, GraphPad Software (San Diego, CA, USA). 

## 3. Results

### 3.1. Ultrasound Treatment Increases the Release of Known Biomarkers in PCa Cell Lines 

**Figure 1:**
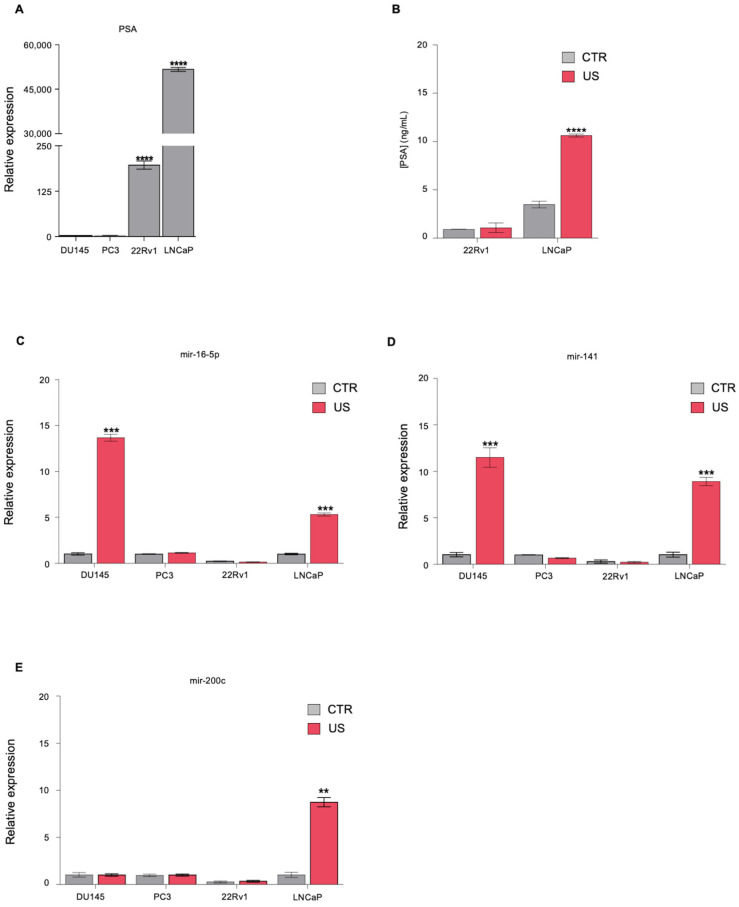
Analysis of biomarkers expression and release in PCa cell lines. (**A**) RT-qPCR showing PSA relative mRNA levels in the indicated PCa cell lines. (**B**) Quantification of PSA release in the supernatant of untreated LNCaP (CTR) or US-treated cells. (**C**–**E**) RT-qPCR showing the levels of (**C**) mir-16-5p, (**D**) mir-141, and (**E**) mir-200c in DU145, PC3, 22Rv1, and LNCaP supernatants from US-treated cells relative to untreated control cells. In all panels, the values denote means ± SD (*n* = 3); statistical significance was calculated by two-tailed Student’s *t*-test. ** *p* < 0.01; *** *p* < 0.001; **** *p* < 0.0001.

A panel of AD (LNCaP, 22Rv1) and AI (DU145, PC3) PCa cell lines was analyzed for the expression of the *PSA* biomarker by RT-qPCR. We found that LNCaP cells expressed the higher levels of *PSA* (fold change [FC]: 51,679.64) compared to the other three cell lines, in which *PSA* expression was either significantly lower (22Rv1, FC: 196.98; PC3, FC: 3.08) or absent (DU145) (Figure 1A).

As circulating PSA detection is routinely used in the clinic for PCa screening, we tested if US treatment could improve the release of this biomarker in the supernatant of LNCaP and 22Rv1 cells, the two cell lines for which we detected measurable levels of PSA mRNA. To this end, both cell lines were treated with the automatized prototype Sonowell^®®^. Consistently with previous evidence, we applied a permeabilization regimen to characterize the molecular features of our PCa lines after US treatment [18]. Medium was collected before and after sonication from the same well to normalize biomarkers values on the same cell number; 10% of duty cycle was chosen to prevent membrane disruption and morphological alterations. Setting experiments identified 1 MHz frequency as the transducer that produced the most homogeneous sonication in the well, based on cellular area coverage analysis, in keeping with the work by D’Souza et al. [17]. PSA levels in the supernatants were detected by ELISA. After 30 min of US treatment, LNCaP cells released measurable levels of PSA in the supernatant, which were found to be almost three times the levels measured for untreated control cells (FC: 2.87) (Figure 1B). Instead, we observed no difference in PSA release between US-treated 22Rv1 and untreated cells. As an additional control of our system and to confirm that the selected parameters were the most efficient in inducing the permeabilization of our cell lines, we further tested our PCa cell lines for the release, before and after US treatment, of three cell-free miRNAs previously isolated by D’Souza and colleagues in LNCaP supernatants after US treatment, i.e., mir-16-5p, mir-141, mir-200c [17]. The release of these selected miRNAs in the supernatant was evaluated through RT-qPCR after total miRNAs extraction. We found increased levels of mir-16-5p and mir-141 in both LNCaP (mir-16-5p, FC: 5.3; mir-141, FC: 2.8) and DU145 (mir-16-5p, FC: 13.6; mir-141, FC: 11.17) supernatants following US treatment, while an increased US-induced release of mir-200c was detected only in LNCaP supernatant (FC: 8.5). No significant increase of miRNA release was detected in PC3 and 22Rv1 supernatants after US treatment (Figure 1C–E). The different response to US treatment observed across PCa cell lines could be due to their diverse morphology, which affects US-induced membrane permeability [24,25,26]. We concluded that US treatment is effective in increasing the extracellular release of PCa-related miRNAs, at least in some cellular settings.

### 3.2. Identification of Novel miRNAs in the Supernatant of PCa Cells following US Treatment 

**Figure 2:**
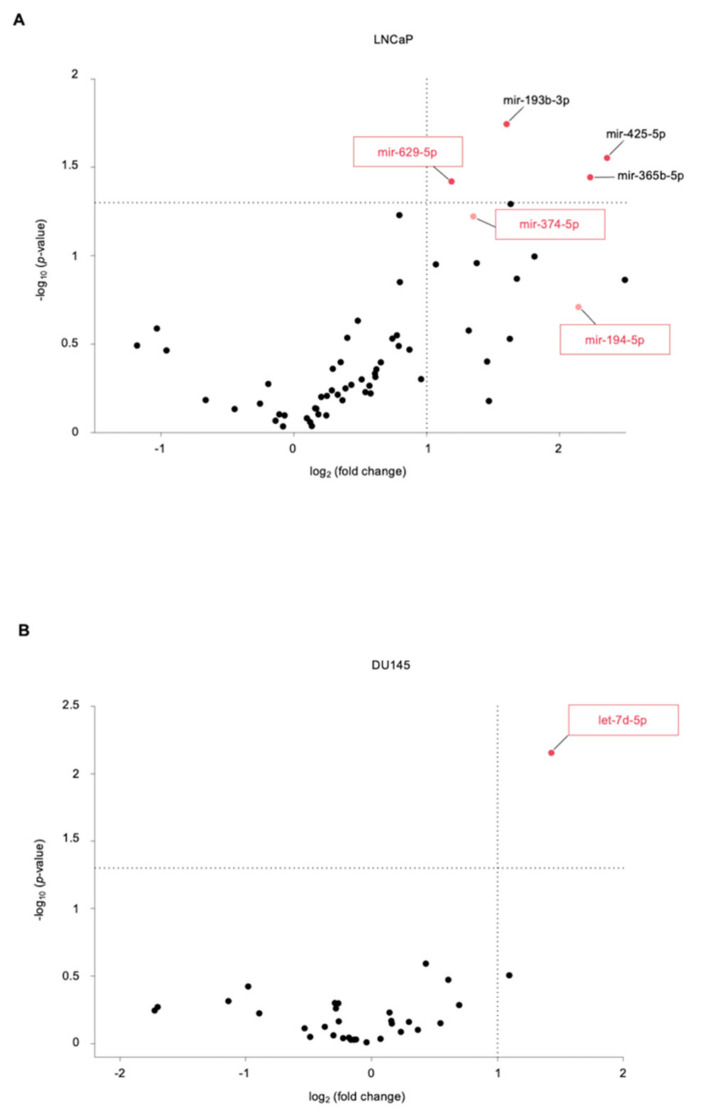
Extracellular miRNAs profiling of US-treated PCa cells. (**A**,**B**) Volcano plots showing the profiles miRNAs released in (**A**) LNCaP or (**B**) DU145 supernatants after US treatment relative to those of untreated control cells, performed by using TaqMan™ Advanced miRNA Human Serum/Plasma Card RT-qPCR array cards. The analyses were performed using Expression Suite software. Reported are the negative log10 *p*-values plotted against the log2 fold change. Dots represent individual miRNAs. Horizontal line, *p* = 0.05; Vertical line, FC = 2. Dark red dots, miRNAs released at significantly higher levels (*p* < 0.05). miRNAs of interest are depicted (framed boxes).

Since US treatment showed to be effective in increasing the concentration of known miRNAs released in the supernatant of LNCaP and DU145 cells, we profiled the miRNAs released by these cells before and after US treatment, with the aim of identifying new potential PCa-related miRNA biomarkers. The miRNA profile analysis was performed by TaqMan™ Advanced miRNA Human Serum/Plasma RT-qPCR array cards. The volcano plots in Figure 2 show the log2FC of all miRNAs detected in LNCaP (Figure 2A) or DU145 (Figure 2B) supernatants after 1 h of US treatment compared to those released from untreated cells over the same time (see also Supplementary Table S1). 

**Figure 3:**
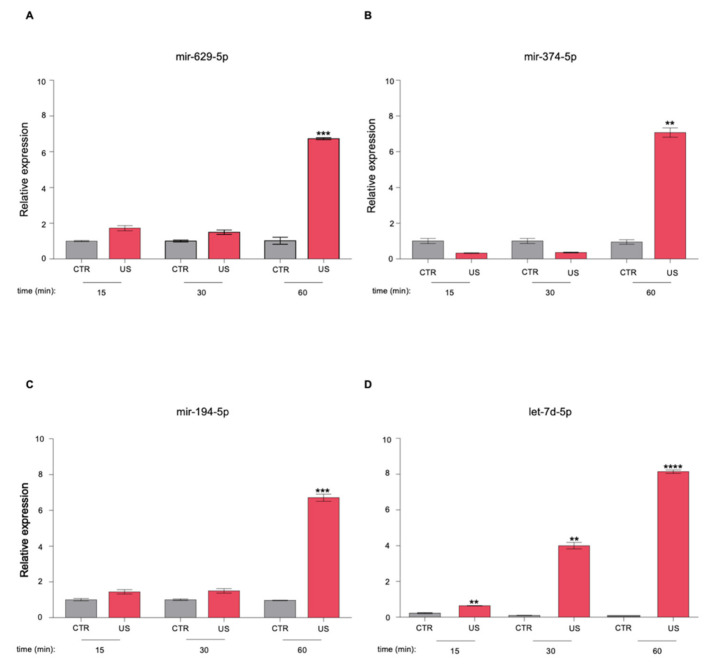
Cellular release kinetics of cell-free miRNAs released from US-treated PCa cells. (**A**–**C**) Single RT-qPCR assay showing cellular release kinetics of (**A**) mir-629-5p, (**B**) mir-374-5p, and (**C**) mir-194-5p in LNCaP supernatant following US treatment for different time periods. (**D**) Single RT-qPCR assay showing cellular release kinetics of let-7d-5p in DU145 supernatants following US treatment for different time periods. In all panels, the expression values are relative to those of untreated control cells. Values denote means ± SD (*n* = 3); statistical significance was calculated by two-tailed Student’s *t*-test. ** *p* < 0.01; *** *p* < 0.001; **** *p* < 0.0001.

Among the 188 miRNAs investigated, we identified 4 miRNAs, whose levels were significantly higher in LNCaP supernatant after US treatment compared to those detected in basal conditions: miR-425-5p (FC: 5.129, *p* = 0.028), miR-365b-3p (FC: 4.698, *p* = 0.036), miR-629-5p (FC: 2.274, *p* = 0.038), and miR-193b-3p (FC: 3.034, *p* = 0.018). Three of them, miR-425-5p, miR-365a-3p, and miR-193b-3p, were already described in the literature to be involved in PCa. In fact, miR-425-5p has been described to be overexpressed in PCa cell lines, where it promotes proliferation, migration, and invasion by targeting forkhead box J3 [27]; miR-365b-3p expression resulted to be expressed at statistically different levels in PCa tissues compared to tissue from patients with prostatic hyperplasia [28]; the silencing of miR-193b-3p through promoter methylation was shown to correlate with more aggressive PCa [29]. Interestingly, we also identified miR-629-5p, which to our knowledge has never been linked to PCa disease (Figure 2A, Supplementary Table S1). Notably, two additional miRNAs, whose supernatant levels were increased more than two-fold following LNCaP US treatment—miR-374a-5p (FC: 2.550, *p* = 0.060) and miR-194-5p (FC: 4.416, *p* = 0.195)—were not described in the literature as PCa-related miRNAs. DU145 profiling showed a significant increase in the release of let-7d-5p (FC:2.694, *p* = 0.007) following US treatment and, although this miRNA family has been related to several cancers [30], no specific roles in PCa have been described for it (Figure 2B, Supplementary Table S1). We investigated the cellular release kinetics of the newly identified miR-629-5p, miR-374a-5p, miR-194-5p, and let-7d-5p by single RT-qPCR assay. The release of miRNAs in the supernatant of LNCaP and DU145 cells was measured after treating the cells with US for 15 min, 30 min, and 1 h. The supernatants of untreated control cells were collected before sonication, following incubation with exosomes-depleted medium for the same time. We observed a significant increase in the release of miR-629-5p, miR-374a-5p, and miR-194-5p in LNCaP supernatant after 1h of US treatment compared to untreated cells (miR-629-5p, FC: 6.5; miR-374a-5p, FC: 7.1; miR-194-5p, FC: 6.4), while no significant increase was observed at earlier time points (Figure 3A–C).

Instead, we observed a time-dependent increase of let-7d-5p release in the supernatant from US-treated DU145 cells compared to untreated cells. This increase in miRNA release was already significant after 15 min of US treatment (FC: 2.8) and augmented when the cells were treated for longer times (30′ treatment, FC: 40; 1h treatment, FC: 81.3) (Figure 3D).

### 3.3. The Newly Identified miRNAs Are Upregulated in the Serum from PCa Patients

**Figure 4:**
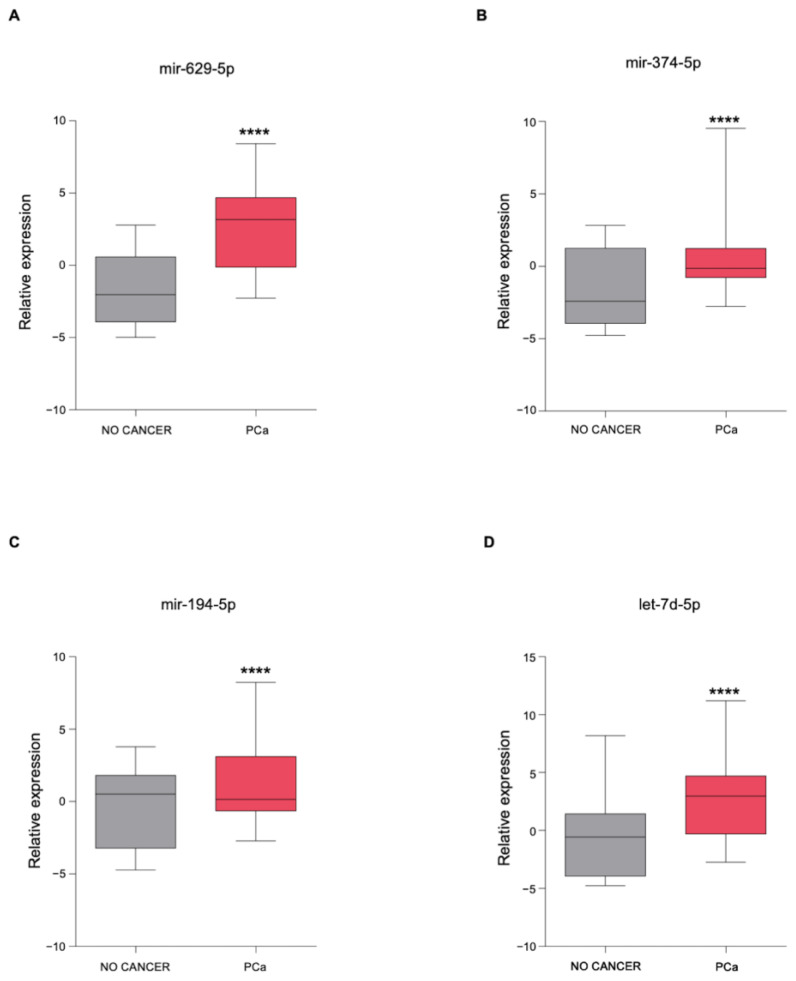
Expression levels of the newly identified miRNAs in the serum from PCa patients. (**A**–**D**) Boxplot showing the expression of (**A**) mir-629-5p, (**B**) mir-374-5p, (**C**) mir-194-5p, and (**D**) let-7d-5p in the serum from control healthy volunteers (*n* = 41) and human PCa patients (*n* = 809) reported in the GSE112264 dataset. Shown in the boxplot are the medians (horizontal lines), 25th–75th percentiles (box outlines), and the highest and lowest values within 1.5× of the inter-quartile range (vertical lines). Statistical significance was calculated by using the two-tailed Mann–Whitney U-test. ** *p*-value < 0.01, **** *p*-value < 0.0001.

To understand the clinical significance of these four miRNAs identified in PCa cell supernatants following US treatment, we analyzed their expression in the serum from non-cancer controls (*n* = 41) and PCa patients (*n* = 809) using the GSE112264 publicly available dataset. This analysis showed that the serum expression of all miRNAs was significantly upregulated in PCa patients compared to controls (Figure 4A–D), suggesting that these miRNAs could be potentially valuable diagnostic biomarkers. 

**Figure 5:**
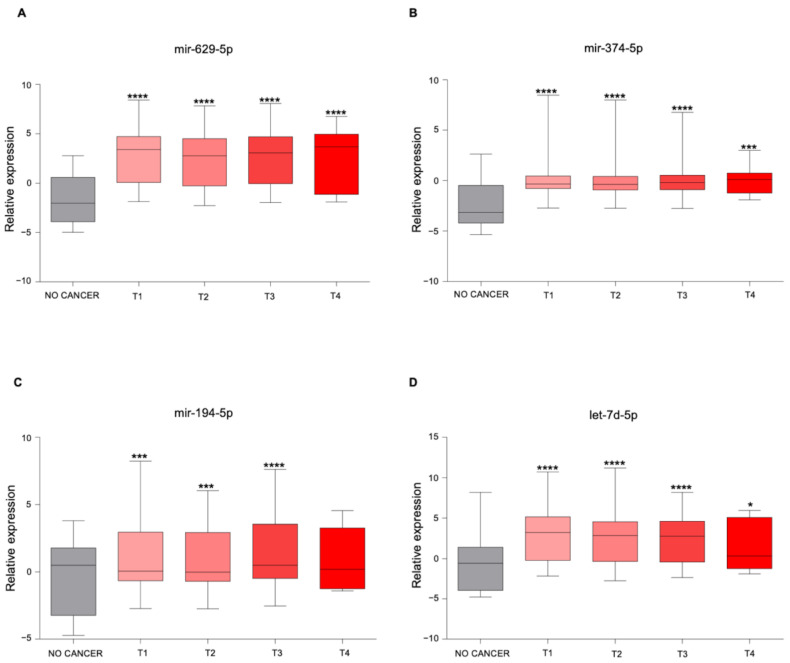
miRNAs expression in the serum from PCa patients stratified according to tumor staging. (**A**–**D**) Boxplot showing the expression of (**A**) mir-629-5p, (**B**) mir-374-5p, (**C**) mir-194-5p, and (**D**) let-7d-5p in the serum from control healthy volunteers (*n* = 41) and human PCa patients from the GSE112264 dataset, stratified according to tumor staging (T1 = 256 samples, T2 = 354 samples, T3 = 183 samples, T4 stage = 16 samples). Shown in the boxplot are the medians (horizontal lines), 25th–75th percentiles (box outlines), and the highest and lowest values within 1.5× of the inter-quartile range (vertical lines). Samples from each tumor stage were compared to control healthy samples by using the two-tailed Mann–Whitney U-test. *, *p*-value < 0.05; ** *p*-value < 0.01; *** *p*-value < 0.001; **** *p*-value < 0.0001. Statistical significance for multiple comparisons was calculated by using the Kruskal–Wallis test. *p*-value < 0.0001 (**A**,**B**,**D**); *p*-value = 0.0146 (**C**).

We also analyzed the serum expression of these miRNAs in PCa patients stratified based on tumor stage. The expression levels of mir-629-5p, mir-374-5p, and mir-194-5p were increased in the serum of PCa patients across all tumor stages compared to control sera (Figure 5A–C), while let-7d-5p mRNA levels were significantly higher only in the serum from patients with T1–T3 PCa disease (Figure 5D).

Thus, all analyzed miRNAs appeared to be released in the serum of PCa patients in the earlier phases of disease (T1–T2 stages), and their levels remained high as the disease progressed to advanced stages (T3–T4 stages). 

### 3.4. In Silico miRNA: Gene Interaction Analysis

**Figure 6:**
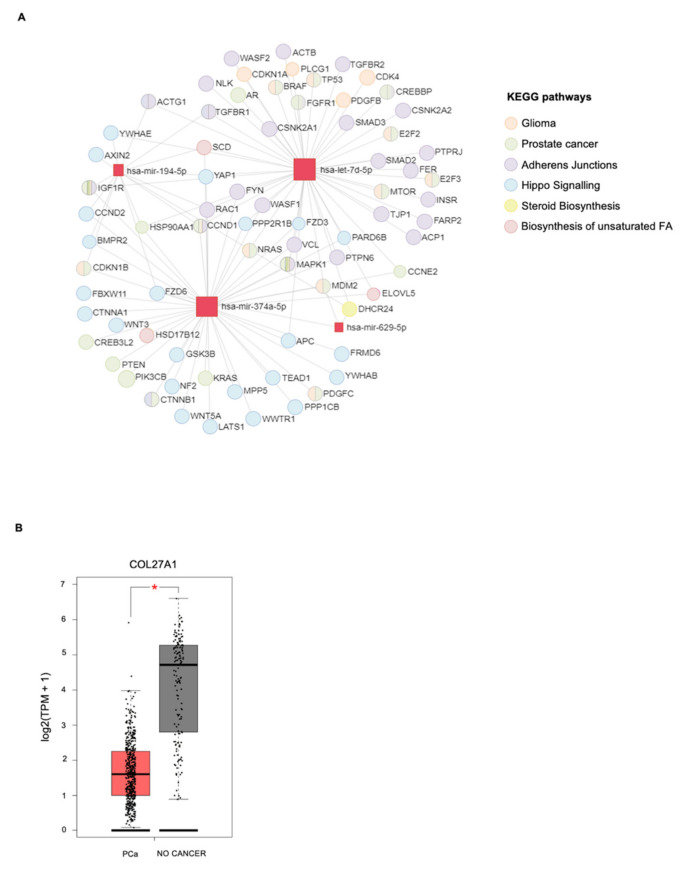
In silico analysis of miRNA–gene interactions. (**A**) Schematic representation of the miRNA–gene network resulting from the enrichment analysis of validated miRNA–gene interactions followed by targeted pathway analysis. A color code was used for grouping genes from the same KEGGS pathway. (**B**) Boxplots showing the mRNA expression of *COL27A1* in tumor samples of PCa (*n* = 492) from the PRAD-TCGA dataset and in normal tissues (*n* = 152) from the TCGA and Genotype-Tissue Expression (GTEx) datasets. Expression analysis was performed and statistical significance was determined using the Gene Expression Profiling Interactive Analysis (GEPIA) web server. TPM, transcripts per million. A–B, * *p*-value < 0.05.

To better characterize the newly PCa-related miRNAs, we conducted an in silico analysis to identify the biological targets of mir-629-5p, mir-374-5p, mir-194-5p, and let-7d-5p, with the aim of defining their roles in PCa development and progression. To this end, DIANA-mirPath 3.0 was used to search out both experimentally validated and putative target genes, by exploiting two different bioinformatic tools: (i) TarBase v7.0, which shows gene targets validated in high-throughput, microarrays, sequencing and proteomic experiments and (ii) microT-CDS, which identifies putative target genes by using an algorithm based on the recognition of positive and negative sets of miRNA Recognition Elements (MREs) located in both the 3’-UTR and the CDS regions. Enrichment analysis of validated miRNA–gene interactions followed by targeted pathway analysis (Figure 6A, Supplementary Table S2) showed that both mir-629-5p and miR-194-5p target the *DHCR24* gene, a central regulator of steroid biosynthesis, which is frequently altered in prostate cancer cells [31]. 

Among the validated gene targets of both miR-194-5p and miR-374a-5p, we also found *CCND2*, *AXIN2*, and *BMPR2*, regulated by the Hippo signaling pathway, involved in stemness and cancer biology. miR-374a-5p seems also to be involved in the regulation of the biosynthesis of unsaturated fatty acids by targeting genes like *ELOVL5*, SCD, and *HSD17B12*, thus reinforcing the well-known correlation between altered lipid metabolism and PCa [32,33,34]. Both miR-374a-5p and let-7d-5p target several genes previously associated with the KEGG PCa pathway. Moreover, we found that let-7d-5p, identified in the supernatant of DU145 following US treatment, regulates genes involved in the glioma-associated KEGG pathway, and this is of interest as DU145 cells were isolated from a brain metastasis of PCa. Indeed, these two KEGG pathways share many genes, such as *CCND1*, which plays a fundamental role in the regulation of cell cycle and proliferation in both glioma and PCa cells and whose deregulation portends worse clinical outcomes [35,36]. let-7d-5p also modulates genes implicated in cell–cell adherens junctions, suggesting a role for this miRNA in regulating epithelial–mesenchymal transition (EMT), a process promoting PCa metastasis and chemoresistance [37]. In addition to validated gene interactors, we searched for putative target genes of our miRNAs of interest. Enrichment analysis of putative miRNA–gene interactions followed by targeted pathway analysis performed by microT-CDS suggested that miR-629-5p could also target the genes for prolactin receptor (PRLR) and AKT serine/threonine kinase 3 (AKT3), involved in the prolactin signaling pathway, whose alteration may contribute to the pathogenesis of PCa (Table 1) [38,39]. 

The same analysis showed that miR-194-5p could be also involved in the regulation of branched-chain amino acid degradation, a metabolic process found to be deregulated in PCa progression (Table 1) [40]. Interestingly, among putative targets of let-7d-5p, we found that *COL27A1*, coding for fibrillar collagen α-1 (XXVII) chain protein and, to our knowledge, never associated with PCa, was the only downregulated gene in PCa patients as reported in The Cancer Genome Atlas (TCGA) compared to controls, suggesting a potential role for this let-7d-5p-target gene in PCa pathogenesis (Table 1, Figure 6B) [41].

## 4. Discussion

The use of circulating miRNA biomarkers for improving cancer diagnosis and monitoring treatment response is showing promise for clinical utility [42]. However, these miRNAs are often present at or below quantifiable limits, especially in early-stage disease [14]. Our study provides evidence that US-mediated sonoporation could be a powerful tool to amplify the release of prognostic miRNAs from both AD and AI PCa cells, in keeping with recent studies showing an increased release of known PCa-related miRNA biomarkers from LNCaP cells [17]. Notably, preliminary data in rat allografts and patients with uterine fibroids showed that US treatment could be effective in stimulating the release of intracellular biomarkers in body fluids, suggesting that this technique could be clinically useful for the earlier detection of cancer lesions [17,18]. In most of the cases in which the tumor site is known, the direct application of US energy on the target tissue would be a feasible option to increase the levels of circulating biomarkers. Indeed, most anatomic sites of cancer lesions, especially superficial organs like liver or breast, are accessible to focused US, with the exception for brain, bone, and lung [18]. Moreover, early lesions or tumors of unknown staging are frequently detected incidentally during routine analysis screening [43] or by techniques performed for an unrelated purpose [44,45]; in this scenario, the on-site application of US energy with the following quantification of released biomarkers could be an additional clinical tool for a more precise diagnosis, along with the screening of canonical biomarkers (e.g., PSA, CEA, CA19-9, etc.). However, it is important to consider that the use of this US-based approach to increase the levels of circulating biomarkers is limited to those cases in which the localization of the tumor site is known, as the lack of a precise target area to treat with US would make this approach unfeasible.

Due to the low predictive value of current detection and prognostic tools, PCa overdiagnosis and overtreatment remains a major challenge. Although several studies identified many miRNAs involved in PCa development and progression and investigated their potential as PCa non-invasive biomarkers for early diagnosis and prediction of metastasis [46,47], validation of multiple biomarkers is crucial for an effective detection and management of cancer disease [48,49]. Interestingly, in this regard, our miRNA profiling of US-treated cell supernatants allowed the identification of four novel PCa-related miRNAs with potential diagnostic and predictive value: mir-629-5p, mir-374-5p, mir-194-5p, and let-7d-5p. Indeed, our results showed that their expression in sera from PCa patients is higher compared to their levels in sera from healthy volunteers. Moreover, we showed that these miRNAs seem to be released in the earlier phases of disease, and their levels remain high as the disease progresses to advanced stages, suggesting that they might be valuable tools to improve early cancer detection and patient management along with the currently used biomarkers. Although their utility as PCa biomarkers awaits stronger clinical confirmation, our study also highlighted the potential of US-based miRNA profiling in preclinical studies as a starting point to identify novel potential biomarkers, whose clinical relevance could be explored, in a second step, by using a targeted analysis of datasets from cancer patients.

Furthermore, our analysis of miRNA–gene interactions showed that they target many well-known PCa-related genes, which are often downregulated in aggressive PCa. Indeed, reduced expression of *DHCR24*, a target of both mir-629-5p and miR-194-5, just like other AR-related genes, in primary PCa specimens correlates with PCa progression and higher risk to develop metastases, since androgens induce differentiation and inhibit the growth of prostate epithelial cells [50]. Downregulation of *CCND2*, *AXIN2*, and *BMPR2*, targeted by miR-194-5p and miR-374a-5p, was also associated with advanced PCa disease. In particular, reduced expression of *CCND2* was found in more aggressive prostate tumors characterized by high Gleason score and elevated PSA levels, suggesting that it could be an indicator of increased risk of relapse [51], while downregulation of *AXIN2* and *BMPR2* was associated with high tumor grade, invasiveness, and recurrence [52,53]. In addition to providing a biological connection between newly identified miRNAs and genes with a well-described role in PCa biology, we also identified *COLA27A1* as a putative target gene of let-7d-5p. We found that *COLA27A1* mRNA expression is lower in PCa tumors compared to normal tissues, suggesting a potential role for this gene in PCa pathogenesis. Clinical studies suggested that tumor collagen content, alignment, and distribution are prognostic factors in different cancers, and specific chains of collagens modulate key processes in cancer progression, acting as either pro- or anti-tumorigenic factors [54,55,56,57,58]. Moreover, a recent study reported that high expression levels of *COL27A1* were associated with a more favorable prognosis in breast cancer, pancreatic ductal adenocarcinoma, and kidney renal clear cell carcinoma [59], suggesting that the silencing of this gene by let-7d-5p could be an adverse event associated with more aggressive disease. Further investigations will determine the precise role of this gene in PCa progression and evaluate the prognostic significance of COL27A1 in this cancer.

## 5. Conclusions

Together, our findings highlight the potential of using US to identify novel cell-free miRNAs released from cancer cells. The identification of new PCa-related cell-free miRNAs may be crucial not only for the development of novel biomarkers but also to uncover novel potential pathogenetic mechanisms involved in PCa biology.

## Figures and Tables

**Table 1:** Enrichment analysis of putative miRNA–gene interactions followed by targeted pathway analysis.

miRNAs	KEGG Pathways	*p*-Value	Target Genes
miR-629-5p	Prolactin signaling pathway	4.27 × 10^−3^	*PRLR* *AKT3*
miR-194-5p	Valine, leucine, and isoleucine degradation	6.99 × 10^−4^	*BCKDHA* *ACADSB* *BCAT1*
let-7d-5p	ECM–receptor interaction	1.07 × 10^−2^	*COL27A1* *COL3A1* *COL1A1* *COL1A2*

## Data Availability

Serum miRNA expression data from the GSE112264 dataset are available at https://www.ncbi.nlm.nih.gov/geo/query/acc.cgi?acc=GSE112264, accessed on 30 May 2020. Gene expression data from the TCGA-PRAD and GTEx datasets are available at http://gepia.cancer-pku.cn/detail.php.

## Supplementary Materials

The following are available online at https://www.mdpi.com/article/10.3390/genes12111726/s1, Table S1: Cell-free miRNAs profiling of US-treated LNCaP and DU145 cells. Table S2: Enrichment analysis of validated miRNA–gene interactions followed by targeted pathway analysis.
