# Supplementary material for: Ultrasound-Based Method for the Identification of Novel MicroRNA Biomarkers in Prostate Cancer
Source: Genes (Basel). 2021 Oct 28;12(11):1726. doi: 10.3390/genes12111726 (PMC8619582; doi:10.3390/genes12111726)
Supplement: Supplementary file 1 [file genes-12-01726-s001.zip › genes-1426349-supplementary.pdf]

SUPPLEMENTARY INFORMATION

SUPPLEMENTARY TABLES

Supplementary Table S1. Cell-free miRNAs profiling of US-treated LNCaP and DU145 cells.

LNCaP			
microRNA	Fold Change	Log2 Fold Change (US treated/untreated)	p-value
hsa-miR-193b-3p-478314_mir*	3.034	1.60122109	0.018
hsa-miR-425-5p-478094_mir*	5.129	2.35867757	0.028
hsa-miR-365b-3p-478065_mir*	4.698	2.23204671	0.036
hsa-miR-629-5p-478183_mir*	2.274	1.18523225	0.038
hsa-miR-30b-5p-478007_mir	3.098	1.63133714	0.051
hsa-miR-374a-5p-478238_mir	2.55	1.35049725	0.060
hsa-miR-28-5p-478000_mir	2.096	1.06763872	0.112
hsa-miR-342-3p-478043_mir	2.595	1.37573454	0.110
hsa-miR-30c-5p-478008_mir	3.509	1.81105995	0.101
hsa-miR-99b-5p-478343_mir	5.627	2.49236596	0.137
hsa-miR-200c-3p-478351_mir	3.2	1.67807191	0.135
hsa-miR-194-5p-477956_mir	4.416	2.14274017	0.195
hsa-miR-148b-3p-477824_mir	2.488	1.31498649	0.265
hsa-miR-2110-477971_mir	3.086	1.62573806	0.295
hsa-miR-339-5p-478040_mir	2.739	1.45364927	0.397
hsa-miR-21-3p-477973_mir	2.764	1.46675762	0.664
hsa-miR-103a-3p-478253_mir	1.733	0.79327165	0.059
hsa-miR-374b-5p-478389_mir	1.737	0.79659775	0.141
hsa-miR-19a-3p-479228_mir	1.395	0.48026512	0.233
hsa-miR-182-5p-477935_mir	1.711	0.77483976	0.282
hsa-miR-195-5p-477957_mir	1.671	0.74071173	0.294
hsa-miR-500a-5p-478309_mir	1.727	0.78826808	0.324
hsa-miR-532-5p-478151_mir	1.826	0.86868677	0.34
hsa-miR-222-3p-477982_mir	1.941	0.95680012	0.499
hsa-miR-320e-478022_mir	1.573	0.65351867	0.401
hsa-miR-25-3p-477994_mir	1.538	0.6210555	0.439
hsa-miR-652-3p-478189_mir	1.526	0.60975496	0.464
hsa-miR-378a-3p-478349_mir	1.529	0.61258841	0.485
hsa-miR-132-3p-477900_mir	1.483	0.5685186	0.543
hsa-miR-221-3p-477981_mir	1.492	0.57724754	0.601

hsa-miR-125b-5p-477885_mir	1.452	0.53804145	0.591
hsa-miR-423-3p-478327_mir	1.424	0.50994915	0.5
hsa-miR-186-5p-477940_mir	1.349	0.43189035	0.537
hsa-miR-200a-3p-478490_mir	1.309	0.3884651	0.563
hsa-miR-34a-5p-478048_mir	1.288	0.36513259	0.657
hsa-miR-29a-3p-478587_mir	1.257	0.32998465	0.611
hsa-miR-93-5p-478210_mir	1.219	0.28569813	0.577
hsa-miR-19b-3p-478264_mir	1.225	0.29278175	0.436
hsa-miR-191-5p-477952_mir	1.276	0.35162833	0.4
hsa-miR-92a-3p-477827_mir	1.321	0.40163047	0.291
hsa-miR-26b-5p-478418_mir	1.188	0.24853484	0.621
hsa-miR-26a-5p-477995_mir	1.153	0.20539251	0.629
hsa-miR-21-5p-477975_mir	1.117	0.15962919	0.729
hsa-miR-20a-5p-478586_mir	1.124	0.16864204	0.733
hsa-miR-29b-3p-478369_mir	1.136	0.18396283	0.79
hsa-miR-484-478308_mir	1.184	0.24366908	0.8
hsa-miR-17-5p-478447_mir	1.07	0.0976108	0.831
hsa-miR-28-3p-477999_mir	1.088	0.12167856	0.873
hsa-miR-125b-5p-477885_mir	1.098	0.13487805	0.918
hsa-miR-301a-3p-477815_mir	0.953	-0.0694519	0.8
hsa-let-7g-5p-478580_mir	0.927	-0.1093588	0.789
hsa-miR-339-3p-478325_mir	0.909	-0.1376478	0.857
hsa-miR-190a-5p-478358_mir	0.945	-0.0816138	0.923
hsa-miR-148a-3p-477814_mir	0.875	-0.1926451	0.531
hsa-miR-24-3p-477992_mir	0.838	-0.2549779	0.686
hsa-miR-324-5p-478024_mir	0.734	-0.446148	0.737
hsa-miR-151a-3p-477919_mir	0.631	-0.6642881	0.655
hsa-miR-505-3p-478145_mir	0.514	-0.9601597	0.343
hsa-miR-130b-3p-477840_mir	0.489	-1.0320936	0.258
hsa-miR-210-3p-477970_mir	0.441	-1.1811494	0.322
**DU145**			
**microRNA**	**FC**	**Log2 FC**	**p-value**
hsa-let-7d-5p-478575_mir*	2.694	1.42974985	0.007
hsa-miR-191-5p-477952_mir	2.135	1.09423607	0.312
hsa-miR-21-5p-477975_mir	1.348	0.4308205	0.256
hsa-miR-24-3p-477992_mir	1.525	0.60880924	0.337
hsa-miR-342-3p-478043_mir	1.62	0.69599381	0.518
hsa-miR-23a-3p-478532_mir	1.46	0.54596837	0.706
hsa-miR-151a-3p-477919_mir	1.29	0.36737107	0.791
hsa-miR-15a-5p-477858_mir	1.228	0.29631056	0.691
hsa-miR-10a-5p-479241_mir	1.175	0.23266076	0.819
hsa-miR-31-5p-478015_mir	1.118	0.16092019	0.71

hsa-miR-29b-3p-478369_mir	1.115	0.15704371	0.678
hsa-miR-93-5p-478210_mir	1.104	0.14274017	0.589
hsa-miR-26a-5p-477995_mir	1.05	0.07038933	0.922
hsa-miR-99b-5p-478343_mir	0.973	-0.0394883	0.978
hsa-miR-652-3p-478189_mir	0.918	-0.1234339	0.932
hsa-miR-155-5p-477927_mir	0.907	-0.1408255	0.935
hsa-miR-181a-5p-477857_mir	0.892	-0.1648844	0.936
hsa-miR-222-3p-477982_mir	0.883	-0.1795147	0.903
hsa-miR-25-3p-477994_mir	0.855	-0.2260037	0.912
hsa-miR-320a-478594_mir	0.811	-0.3022262	0.868
hsa-miR-423-3p-478327_mir	0.835	-0.2601519	0.684
hsa-let-7g-5p-478580_mir	0.822	-0.2827897	0.549
hsa-miR-125b-5p-477885_mir	0.833	-0.2636116	0.503
hsa-miR-29a-3p-478587_mir	0.817	-0.291592	0.501
hsa-miR-186-5p-477940_mir	0.773	-0.3714597	0.751
hsa-miR-532-5p-478151_mir	0.713	-0.488026	0.893
hsa-miR-200c-3p-478351_mir	0.692	-0.5311561	0.77
hsa-miR-193b-3p-478314_mir	0.539	-0.8916428	0.597
hsa-miR-221-3p-477981_mir	0.507	-0.9799423	0.378
hsa-miR-502-3p-478348_mir	0.455	-1.1360615	0.485
hsa-miR-28-3p-477999_mir	0.308	-1.6989977	0.536
hsa-miR-500a-5p-478309_mir	0.303	-1.7226103	0.568

*Significantly up-released miRNAs, $p < 0.05$

Supplementary Table S2. Enrichment analysis of validated miRNA:gene interactions followed by targeted pathway analysis.

**miRNAs**	**miR-629-5p**	**miR-374a-5p**			**miR-194-5p**		**let-7d-5p**		
**KEGG pathways**	Steroid biosynthesis	UFA biosynthesis	Hippo pathway	Prostate cancer	Hippo pathway	Steroid biosynthesis	Adherens junction	Glioma	Prostate cancer
***p*-value**	4.63E-05	3.08E-08	3.085E-08	2.21E-02	2.65E-04	5.30E-04	1.94E-06	8.64E-03	2.37E-02

**Target Genes**	DHCR24	ELOVL5	GSK3B	NRAS	TGFBR1	DHCR24	ACTB	BRAF	BRAF
		SCD	YAP1	PIK3CB	YAP1		CSNK2A2	CDK4	E2F2
		HSD17B12	APC	CDKN1B	YWHAE		TGFBR1	E2F2	NRAS
			NF2	IGF1R	CCND2		WASF1	NRAS	HSP90AA1
			WNT5A	KRAS	ACTG1		SMAD2	IGF1R	IGF1R
			CCND2	CCND1	CCND1		ACTG1	TP53	TP53
			FZD6	CTNNB1	AXIN2		SMAD3	PLCG1	AR
			WNT3	CCNE2	BMPR2		IGF1R	CCND1	CCND1
			YWHAB	PDGFC			VCL	E2F3	CCNE2
			WWTR1	CREB3L2			TJP1	PDGFB	E2F3
			FZD3	PTEN			FYN	CDKN1A	PDGFB
			MPP5	MAPK1			ACP1	MTOR	CDKN1A
			CCND1	MDM2			NLK	MAPK1	MTOR
			CTNNB1				PTPN6	MDM2	FGFR1
			CTNNA1				WASF2		MAPK1
			FRMD6				FER		CREBBP
			TEAD1				CSNK2A1		MDM2
			LATS1				FARP2		
			FBXW11				PTPRJ		
			PPP2R1B				RAC1		
			PARD6B				INSR		
			BMPR2				FGFR1		
			PPP1CB				MAPK1		
							CREBBP		
							TGFBR2		
